# Rationale and design of PASSAT — patients’ satisfaction with local or general anaesthesia in video-assisted thoracoscopic surgery: study protocol for a randomised controlled trial with a non-randomised side arm

**DOI:** 10.1186/s13063-019-3190-1

**Published:** 2019-02-27

**Authors:** Thomas Galetin, Pascal Bretzke, Alberto Lopez-Pastorini, Mark Schieren, Aris Koryllos, Nils Kosse, Jost Schnell, Jerome M. Defosse, Frank Wappler, Erich Stoelben

**Affiliations:** 1 University Witten/Herdecke, Department of Thoracic Surgery, Alfred-Herrhausen-Str. 50, Witten, D-58448 Germany; 2 University Witten/Herdecke, Department of Anaesthesiology and Intensive Care Medicine, Alfred-Herrhausen-Str. 50, Witten, D-58448 Germany; 3 University Witten/Herdecke, Medical Centre Cologne-Merheim, Department of Thoracic Surgery, Ostmerheimer Str. 200, Cologne, D-51109 Germany; 4 University Witten/Herdecke, Medical Centre Cologne-Merheim, Department of Anaesthesiology and Intensive Care Medicine, Ostmerheimer Str. 200, Cologne, D-51109 Germany; 5 Sana IT Services GmbH, Burger Straße 211, Remscheid, 42859 Germany

**Keywords:** Patients’ satisfaction, VATS, Thoracic surgery, Psychometrically validated questionnaire, Local anaesthesia, General anaesthesia, Cost effectiveness

## Abstract

**Background:**

Although general anaesthesia (GA) with one-lung ventilation is the current standard of care, minor thoracoscopic surgery, i.e. treatment of pleural effusions, biopsies and small peripheral pulmonary wedge resections, can also be performed using local anaesthesia (LA), analgosedation and spontaneous breathing. Whilst the feasibility and safety of LA have been demonstrated, its impact on patient satisfaction remains unclear. Most studies evaluating patient satisfaction lack control groups or do not meet psychometric criteria. We report the design of the PASSAT trial (PAtientS’ SATisfaction in thoracic surgery – general vs. local anaesthesia), a randomised controlled trial with a non-randomised side arm.

**Methods:**

Patients presenting for minor thoracoscopic surgery and physical eligibility for GA and LA are randomised to surgery under GA (control group) or LA (intervention group). Those who refuse to be randomised are asked to attend the study on the basis of their own choice of anaesthesia (preference arm) and will be analysed separately. The primary endpoint is patient satisfaction according to a psychometrically validated questionnaire; secondary endpoints are complication rates, capnometry, actual costs and cost effectiveness. The study ends after inclusion of 54 patients in each of the two randomised study groups.

**Discussion:**

The PASSAT study is the first randomised controlled trial to systematically assess patients’ satisfaction depending on LA or GA. The study follows an interdisciplinary approach, and its results may also be applicable to other surgical disciplines. It is also the first cost study based on randomised samples. Comparison of the randomised and the non-randomised groups may contribute to satisfaction research.

**Trial registration:**

German Clinical Trials Register, DRKS00013661. Registered on 23 March 2018.

**Electronic supplementary material:**

The online version of this article (10.1186/s13063-019-3190-1) contains supplementary material, which is available to authorized users.

## Background

Video-assisted thoracoscopic surgery (VATS) is the current standard of care for most thoracic operations, ranging from the management of pleural effusions to extended anatomic and oncological radical resections. The standard anaesthetic approach is general anaesthesia (GA) with double-lumen tube and one-lung ventilation (OLV). By inducing a controlled collapse of the non-dependent lung, OLV provides optimal access to the surgical field, especially to “hidden” sites like the mediastinal face of the lung. GA improves surgical conditions, as neuromuscular blockade reduces unwanted diaphragmatic movements, and profound anaesthesia suppresses coughing when central, peribronchial structures are prepared. This setup has enabled modern thoracic surgery since the 1960s [1, 2].

Despite these advantages, GA also has some adverse effects. The collapse of the non-dependent lung and its ventilation-perfusion mismatch are proinflammatory on the alveolar level: lesion of the glycocalyx, increased alveolo-capillary permeability, surfactant dysfunction and alveolar oedema may contribute to the development of acute respiratory distress syndrome (ARDS) [3–6]. Some muscle relaxants can cause an exacerbation of chronic obstructive pulmonary disease (COPD) via histamine release and impaired bronchial constriction [7]. Especially in elderly patients, GA is associated with a high rate of postoperative cognitive dysfunction (POD; 19−30*%*) such as delirium [8–10]. POD leads to longer hospital stays, higher demands for nursing care, medication and mobilisation therapy and significantly higher costs [11].

However, there is no need for profound anaesthesia and OLV in many surgical indications. The minimised access via VATS is predestined for local anaesthesia (LA), i.e. intercostal block, accompanied by analgosedation. The respective indications are the treatment of pleural effusions, biopsies and small peripheral wedge resections. A number of recent studies have evaluated the role of LA in VATS [1, 12–17]. In combination with a laryngeal mask airway and intraoperative vagal block, even extended anatomic lung resections are possible [18, 19]. Of course, this extended approach of “monitored anaesthesia care” (MAC) is even more complex than the standard of care [20].

Indications for LA or GA are mostly determined by the physician, not the patient [21]. The decision may be influenced by available staff resources, the surgeon’s convenience during the operation or financial interests. As LA is considered to be cheaper than GA [22–26], cost pressures in health care systems may lead to increasing use of local or regional anaesthesia. Most existing cost estimations are not based on randomised controlled trials (RCTs), and they arise from different health care systems. Cost studies applicable to the German health care system, which is based on diagnosis-related groups, are scarce.

Although an increasing number of operations in various surgical specialties are being performed on conscious and spontaneously breathing patients, little is known about the influence of the type of anaesthesia on patient satisfaction. Indeed, patient satisfaction is the most neglected aspect in the currently published research on LA vs. GA.

One reason may be that “satisfaction” is challenging to investigate, as there are innumerable influences, for example the patients’ expectations, the treatment outcome, the perceived care and attention of the staff and many other known and unknown variables [27]. Hence, simply asking “Were you satisfied?” is insufficient to measure the differential impact of anaesthesia. A reliable evaluation of patient satisfaction requires multimodal strategies, including the use of validated questionnaires. Two thorough reviews found more than 3000 studies claiming to evaluate patient satisfaction; however, only 73 used instruments which met valid psychometric criteria [28, 29]. Some of these were constructed for special clinical settings, for example paedriatics, obstetrics or local, regional or general anaesthesia, and they were validated for different languages. Among these, only the “ANP” (“Anästhesiologische Nachbefragungsbogen für Patienten”, i.e. “Anaesthesiologic Questionnaire for Patients”) is suitable for our purpose [30].

Apart from the aforementioned physiological and financial aspects, patient satisfaction should be equally considered in clinical decision making. Hence, it must be investigated in a systematic, i.e. randomised controlled manner. PASSAT is the first RCT on this topic using a validated questionnaire and calculating a sufficient sample size, not only for thoracic surgery, but for surgical disciplines in general.

## Materials and methods

### Study objective

The primary objective of PASSAT is to assess the impact of the anaesthetic technique on the satisfaction of patients undergoing minor VATS. The patients are randomised to receive either GA with OLV or LA with analgosedation and spontaneous breathing. Secondary objectives are complication rates, intra- and postoperative carbon dioxide partial pressure, cost and cost effectiveness (Table 1). Furthermore, patients who refuse randomisation are asked to attend the non-randomised, preference-based side arm of the study. Data from the randomised and non-randomised groups will be compared.

**Table 1:** Endpoints

1	Primary endpoint: patients’ satisfaction on the anaesthesia related satisfaction scale of the ANP questionnaire
2	Secondary endpoints:
	∙ Complication rates: systemic inflammatory response syndrome (SIRS), postoperative mechanical ventilation, atrial fibrillation, cardiopulmonary resuscitation (CPR)/ cardiac infarction, air leakage > 7 days, re-operation, pneumonia, new chest tube after surgery, pleural empyema, relevant postoperative bleeding, neurological complications, wound infection, chylothorax, renal failure, heparin-induced thrombopenia, pericardial effusion, pulmonary infarction, pulmonary embolism, paresis of the recurrent laryngeal nerve, abdominal complication, peripheral vascular complication, other complication (to be specified), death
	∙ Intra- and postoperative carbon dioxide tension: (1) arterial blood gas analysis: awake, at wound closure, 30 and 60 min after wound closure; (2) end-tidal capnometry: continuous recording during the operation and for 60 min after; (3) peak pCO_2_ during surgery; (4) time to recovery from peak to baseline pCO_2_
	∙ Direct medical costs: expenses for surgery related personnel (physicians and nurses), medication and medical devices, inpatient care, length of hospital stay

### Study design

**Fig. 1:**
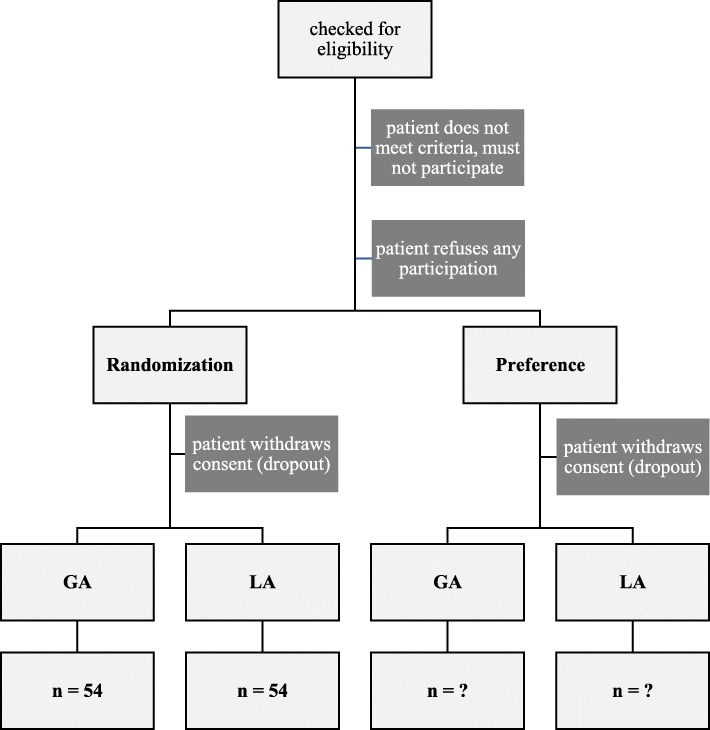
Consolidated Standards of Reporting Trials (CONSORT) flow chart of PASSAT trial

PASSAT is a monocentric, unblinded, parallel, RCT carried out at the Department of Thoracic Surgery of Private University Witten/Herdecke, located at the Lungclinic Cologne-Merheim, which is one of the largest departments of thoracic surgery in Germany. The trial contains an additional non-randomised, preference-based side arm (Fig. 1).

The protocol is described according to the Standard Protocol Items: Recommendations for Interventional Trials (SPIRIT) Checklist for clinical trials (see Additional file 1).

### Patients

Patients with an indication for video-assisted thoracoscopic management of pleural or pericardial effusion, intrathoracic lymph node biopsies or peripheral pulmonary wedge resection are eligible (Table 2). Currently, about 260 VATS for those minor indications are performed at the investigating clinic per year. Operations which are more extensive need more elaborate forms of anaesthesia than simple LA and analgosedation and are therefore not subject to the trial.

**Table 2:** Key inclusion criteria

∙ Indication for VATS for
1 Partial pleurectomy
2 Talcum pleurodesis
3 Implantation of permanent pleural catheter
4 Hilar or mediastinal lymph node biopsies
5 Peripheral pulmonary wedge resection
6 Pericardial effusions
∙ Age ≥ 18 years
∙ Eligibility for LA as well as GA
∙ Informed consent

The exclusion criteria ensure that the planned operations will be safely performed in LA with analgosedation (Table 3).

**Table 3:** Key exclusion criteria

∙	Systematic lymphadenectomy
∙	Expected difficult airway
∙	Emergent operation
∙	Severe coagulation disorders (partial thromboplastin time, PTT > 40 s; international normalised ratio, INR > 1.5)
∙	Previous ipsilateral radiation or surgery
∙	COPD with severe impairment of pulmonary function: diffusing capacity of the lung for carbon dioxide (DLCO)/alveolar volume (AV) < 30%, forced expiratory volume in 1 s (FEV_1_) < 30%
∙	Pregnancy and lactation

Patients do not receive any compensation.

### Randomisation and preference arms

Eligible patients are randomised with variable block size without stratification to the control (GA) or test group (LA) as shown in Fig. 1. Patients are allocated using the built-in randomisation module of the electronic data capture (EDC) software (see the following section on “Data management and confidentiality”).

Patients who refuse to be randomised are asked to attend the preference arm. They may choose their form of anaesthesia. The preference arm is not limited to a certain sample size. Recruitment ends when the randomised arms achieve the calculated sample size.

### Data management and confidentiality

All data are entered into a web-based EDC software which is used for data management and is fully compliant with the principles of good clinical practice and all relevant standards of data handling and protection [31]. The EDC contains modules to randomise patients after checking the inclusion and exclusion criteria and to report adverse events. All actions such as edits and changes in the study structure, data collection and study management are saved in the audit trail. Data can only be archived, not deleted. The platform and data are hosted on European servers, and the data are archived for 15 years. The original paper-based research forms will be archived for 10 years. Access to the study data will be restricted, and all appropriate measures will be taken to preserve confidentiality of medical and personal information. Due to the nature of the intervention, blinding is not possible. The data are captured by a study nurse and analysed by the investigators confidentially and pseudonymously.

### Anaesthetic and surgical procedures

All patients are informed in detail about the operation (procedure, risks and alternatives) and the purpose of the study by the surgeon. If the patient agrees, he is randomised to either LA or GA, and the allocation is communicated to the anaesthesiologist, who then informs the patient in detail about the planned anaesthesia.

The following steps apply to both groups: All patients receive midazolam p.o. 1 h before surgery. The surgeon injects mepivacaine at the site of trocars and into the intercostal space in the posterior axillary line. By the end of the operation, piritramide is administered intravenously.

In the LA group, analgosedation via remifentanil and, if necessary, propofol is administered and monitored clinically by the anaesthesiologist and the anaesthesiologic nurse; instrument-based sleep monitoring is not used.

In the GA group, a standard total intravenous anaesthesia with remifentanil, propofol and rocuronium is administered by the anaesthetist and an anaesthesiologic nurse. Neuromuscular monitoring (i.e. train-of-four) is used to assess the level of muscle relaxation.

VATS will be performed via two or three trocars, placed in one or two intercostal spaces, after the trocar site is infiltrated with mepivacaine. If the surgeon needs a mini-thoracotomy to complete the operation or if the operation in LA needs to be interrupted, the patient will be analysed within the original group, according to an intention-to-treat analysis. Interruption criteria are summarised in Table 4.

**Table 4:** Criteria for interruption of the operation under LA

∙	Surgical complications: major bleeding, severe adhesions, large tumours, unexpected long operation time
∙	Severe hypoxaemia, pO_2_ < 60mmHg
∙	Severe hypercapnia, pCO_2_ > 80mmHg
∙	Haemodynamic instability
∙	Circumstances hindering the surgeon from preparation: persistent coughing, extensive diaphragmatic movements, insufficient collapse of the lung

The intraoperative monitoring tests are identical in either group: blood pressure, electrocardiography, peripheral O_2_ saturation, end-expiratory carbon dioxide, blood gas analysis. Postoperatively, all patients are monitored at the postoperative care unit before returning to the ward.

### Psychometric evaluation and measurements

All patients complete the Hospital Anxiety and Depression Scale (HADS [32]) and the Pain Sensitivity Questionnaire (PSQ [33]) preoperatively, since higher grades of anxiety, depression or pain sensitivity are known to impair the patient’s satisfaction with medical treatments [34, 35]. Postoperatively, all patients answer the Anaesthesiologic Questionnaire for Patients (ANP [30]) and the German version of the patients’ satisfaction with anaesthesia questionnaire of Dr Capuzzo [36].

The ANP consists of several symptom and three different satisfaction scores — satisfaction with recovery, with general perioperative care and with anaesthesia — of which the latter represents our primary endpoint.

After the operation, the surgeon and the anaesthetist each rate their satisfaction with the patient’s coughing and pressing, the collapse of the lung and the general feasibility of the procedure on numeric rating scales (NRSs) from 0 to 10.

**Fig. 2:**
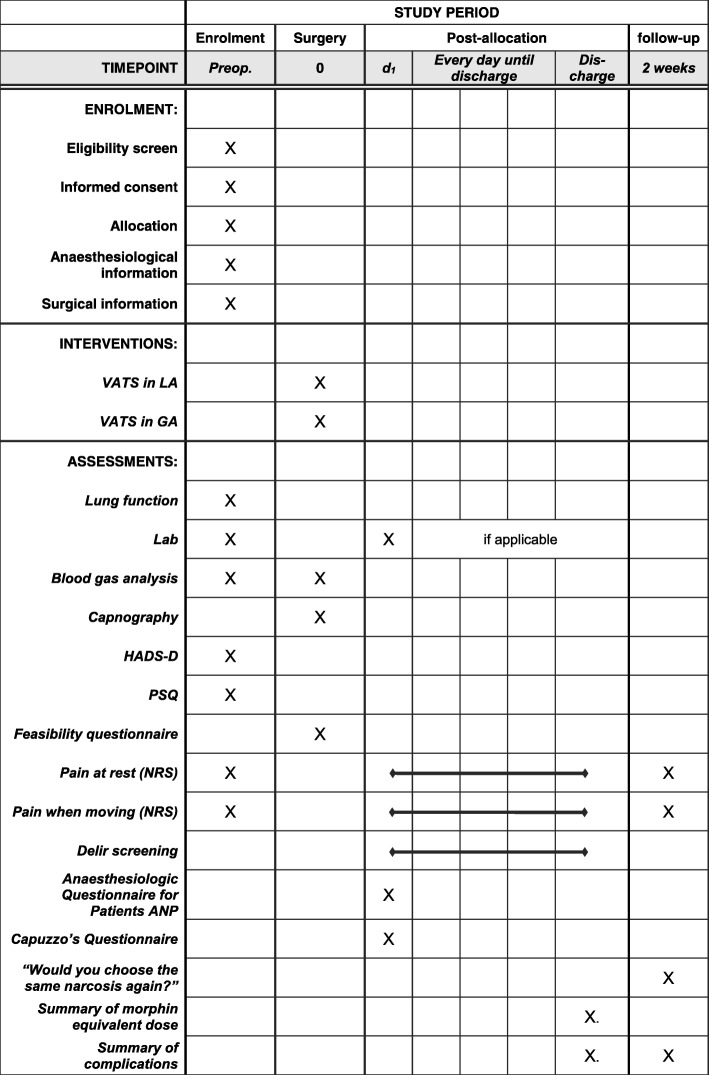
SPIRIT-conforming timeline of study procedures and outcome assessment

Postoperatively, a screening for delirium is performed daily by the ward nurses, using the Nursing Delirium Screening Scale (Nu-DESC [37]) and the Confusion Assessment Method (CAM [38]) which together can detect delirium with a sensitivity of 83−100*%* and a specificity of 81−89*%* [39]. Pain is recorded daily using an NRS from 0 to 10. All complications will be recorded. Standard measurements such as operation time, hospital length of stay, drainage treatment time, serum C-reactive protein and analgesic consumption will be documented as part of the clinical routine (Fig. 2).

### Hypothesis and sample size calculation

The null hypothesis is that patients with LA are as satisfied with anaesthesia according to the “satisfaction with anaesthesia” score of the ANP as those with GA. The alternative hypothesis is that satisfaction in both groups is not equal. The score is noted on an ordinal scale from 0 to 3. The mean satisfaction in the evaluation study was *μ*=2.58 with a standard deviation of *σ*=0.54 [30]. A difference of satisfaction of *ε*=0.32 point is defined as clinically relevant, resulting in a mid-scale effect size *ε*/*σ*=0.59. With a significance level of *α*=5*%*, a power of (1−*β*)=80*%* and an allocation rate of 1:1, we need

$$ n=\left(1+\frac{1}{1} \right) * \left(\sigma * \frac{Z_{(1-{\alpha}/{2})} + Z_{(1-\beta)} }{\epsilon}\right)^{2}=45 $$

patients in each randomised group [40]. Since the ANP satisfaction scale is ordinal, we correct the sample size by +10%. Furthermore, we expect a dropout rate of 10%. In summary, 2∗45∗1.1∗1.1=108 patients will be included in the randomisation group of the study.

Statistical analyses will be performed using the statistical language R. Data will be presented using descriptive statistics such as frequency or mean and standard deviation. Individual changes from baseline to the end of treatment will be analysed if appropriate. After checking for normal distribution, the primary endpoint will be tested with Student’s *t* test or the Mann-Whitney *U* test, respectively. There will not be an interim analysis for the primary endpoint. Multivariate analysis will be performed to identify independent variables which then can be the focus of further investigations.

### Approval, end of study, registration

The study is approved by the Ethics Committee of Private University of Witten/Herdecke, Germany. This study is in compliance with the Helsinki Declaration and with the International Conference on Harmonisation – Good Clinical Practice. In case of necessary protocol amendments, the amendment will be implemented after approval by the ethics committee. The study ends regularly when the planned sample size is achieved. It will be discontinued prematurely if the risk-benefit ratio indicates potential patient harm or the study proves to be impracticable. The actual trial status is publicly visible on the German Clinical Trials Register website. The results will be published.

## Discussion

PASSAT is a prospective RCT with a non-randomised side arm which compares the procedure-related patients’ satisfaction with thoracoscopic operations performed in either local anaesthesia, analgosedation and spontaneous breathing (intervention group) or in general anaesthesia with OLV (control group). Despite GA with OLV being the most common anaesthetic technique used in thoracic surgery, the LA and analgosedation technique is a suitable alternative for the surgical management of numerous indications such as pleural diseases and small peripheral resections. However, the awareness of being brought into the operating room, preparation of the surgical field, the surgery itself and of being brought to the postoperative care unit may be a stressor for the patient [41], depending on the staff’s attention toward the patient and her or his stress coping resources.

Furthermore, one has to be aware of intraoperative hypercapnia due to iatrogenic pneumothorax and paradoxical diaphragm movement [16, 19, 42]. To prevent hypercapnia, patients with severely impaired lung function are recommended to be operated on using GA with controlled mechanical ventilation. However, the existing recommendations—which are respected in this study—are based on expert opinions [12, 19]. A single small study evaluated intraoperative carbon dioxide tension during thoracoscopy in LA (*n*=16), but without reporting the patients’ preoperative lung function [43]. To ensure patients’ safety, we will use intraoperative airway capnography in both groups. The data will be continuously recorded and analysed depending on the preoperative pulmonary function.

Since procedures in LA are believed to be cheaper than those in GA but cost estimations from the German health care system are lacking, we will accurately record and compare the costs, including material and personal resources.

Decision making is a process that involves not only medical and economic aspects, but also the patient’s perspective. However, the patient’s perception and procedure-related satisfaction have not yet been properly investigated. Most studies on surgery in LA do not assess patients’ satisfaction at all or do so in an insufficient manner. Satisfaction is influenced by innumerable variables; thus properly designed questionnaires which meet psychometric criteria are required.

Most studies concentrate on feasibility or safety of operations in LA instead of satisfaction. The only RCT comparing local, spinal and general anaesthesia for the same procedure (inguinal hernia repair, *n*=25 each) reported higher satisfaction in the LA group [44]. These results, however, should be interpreted with caution, as patient satisfaction was assessed unspecifically 6 weeks after surgery. Considering previously mentioned confounders, the differential impact of the anaesthetic approach remains unclear. Furthermore, the trial was not designed to investigate satisfaction as the primary endpoint.

There is only a single comparative, but non-randomised, trial using psychometrically valid methods (the ANP), in which patients with Lichtenstein’s operation chose their anaesthesia according to their personal preference [45]. Patients were not randomised to GA or LA in order to give them a feeling of control. This results in a high risk for bias; hence, it is not surprising that there was no difference in satisfaction between both groups.

A random selection of the anaesthetic technique may be unacceptable for some patients. Instead of being excluded from the study, these patients are asked to participate in the non-randomised preference-based arm, which may provide insights into the influence of self-control on satisfaction. However, this depends on the resulting sample size, which is not predefined for the non-randomised arm.

There are about 37,000 VATS performed for the aforementioned indications in Germany per year [46]. Apart from thoracic surgery, the expected results may also be applicable to other surgical disciplines. Hence, we consider the trial’s subject to be highly relevant.

In summary, PASSAT will assess the impact of local or general anaesthesia on patients’ satisfaction for the first time by means of an RCT. The trial follows an interdisciplinary approach involving surgery, anaesthesia, sociology and economics. Data are expected to be available in 2020.

## Trial status

The trial has been recruiting patients since June 2018. As of December 2018, 27% of the planned sample size has been enrolled.

## Additional file

Additional file 1 Standard Protocol Items: Recommendations for Interventional Trials (SPIRIT) 2013 checklist: recommended items to address in a clinical trial protocol and related documents. (DOCX 45 kb)

## Abbreviations

ANP: Anaesthesiologic Questionnaire for Patients
ARDS: Acute respiratory distress syndrome
AV: Alveolar volume
CAM: Confusion assessment method
CONSORT: Consolidated Standards of Reporting Trials
COPD: Chronic obstructive pulmonary disease
CPR: Cardiopulmonary resuscitation
DLCO: Diffusing capacity of the lung for carbon monoxide
EDC: Electronic data capture
FEV_1_: Forced expiratory volume in 1 s
GA: General anaesthesia
HADS-D: Hospital Anxiety and Depression Scale - German version
INR: International normalised ratio
LA: Local anaesthesia
MAC: Monitored anaesthesia care
NRS: Numeric rating scale
Nu-DESC: Nursing Delirium Screening Scale
OLV: One-lung ventilation
PASSAT: PAtientS’SATisfaction in thoracic surgery
POD: Postoperative cognitive dysfunction
PSQ: Pain Sensitivity Questionnaire
PTT: Partial thromboplastin time
RCT: Randomised controlled trial
SPIRIT: Standard Protocol Items: Recommendations for Interventional Trials
VATS: Video-assisted thoracoscopic surgery
